# Supplementary material for: Parkinson’s disease-associated ATP13A2/PARK9 functions as a lysosomal H+,K+-ATPase
Source: Nat Commun. 2023 Apr 20;14:2174. doi: 10.1038/s41467-023-37815-z (PMC10119128; doi:10.1038/s41467-023-37815-z)
Supplement: Supplementary file 1 — Supplementary Information [file 41467_2023_37815_MOESM1_ESM.pdf]

Supplementary Fig. 1

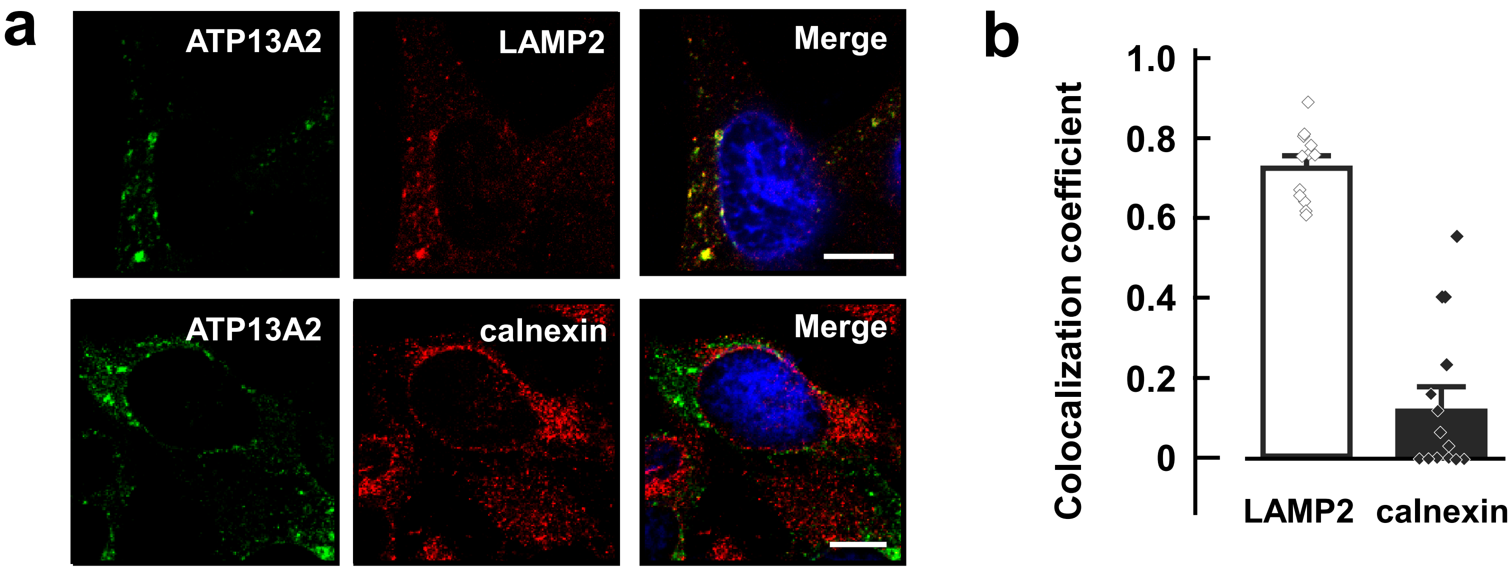

Supplementary Fig. 1 Expression of exogenous ATP13A2 in HEK293 cells. a Immunocytochemistry using anti-Xpress-tag (ATP13A2; green), anti-LAMP2 (red), and anti-calnexin (red) antibodies in the ATP13A2-transfected cells. DNA in the nucleus was visualized with DAPI (blue). Scale bars, 10 µm. **b** Colocalization coefficient of ATP13A2 with LAMP2 or calnexin was calculated with Pearson correlation coefficient analysis. (*n* = 12-14 areas in three independent experiments). All data are presented as mean ± SEM. Source data are provided as a source data file.

Supplementary Fig. 2

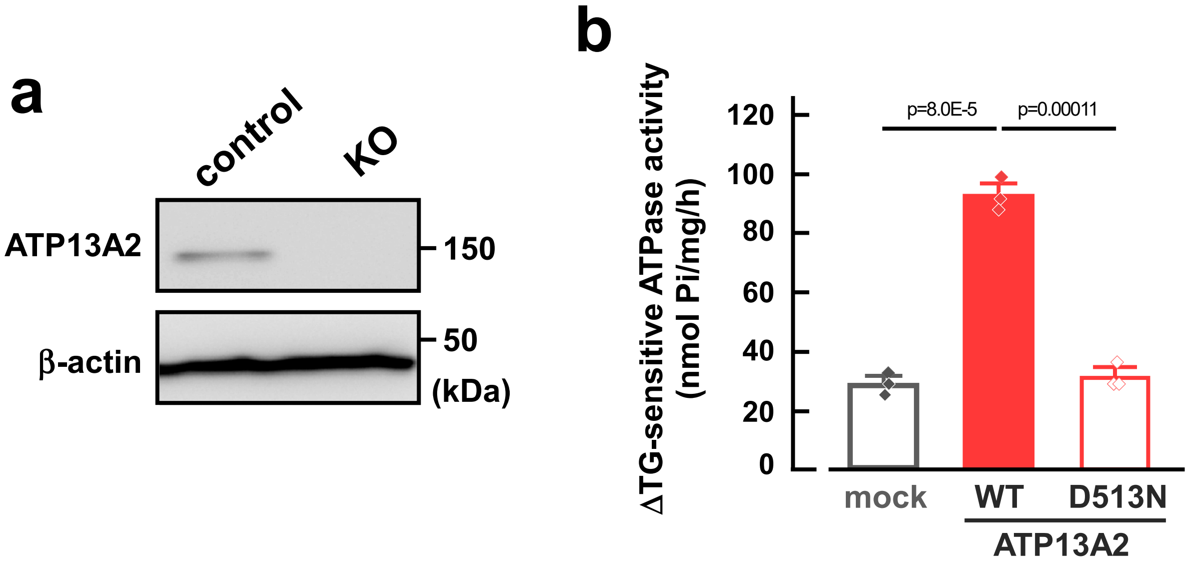

Supplementary Fig. 2 Exogenous ATP13A2 activity in the ATP13A2-knockout HEK293 cells. a Expression of ATP13A2 and β-actin in the ATP13A2-knockout (KO) HEK293 cells. Typical Western blot images in three independent experiments are shown. **b** TG-sensitive ATPase activities in the ATP13A2 WT-, D513N- and mock-transfected KO cells. (n = 3 independent replicates). The basal TG-sensitive ATPase activity in the mock-transfected KO cells (30 ± 2 nmol/mg/h) was smaller than in the mock-transfected control cells (39 ± 3 nmol/mg/h; Fig. 1b). Statistical significance was determined by two-tailed unpaired Student's t-test. All data are presented as mean ± SEM. Source data are provided as a source data file.

Supplementary Fig. 3

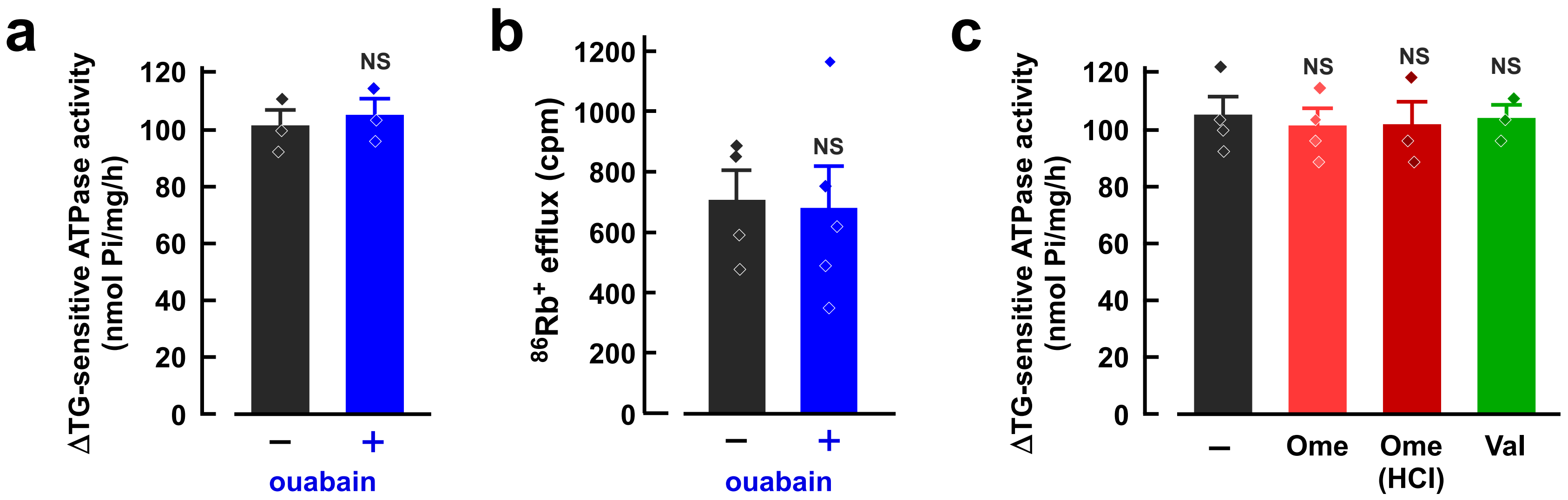

Supplementary Fig. 3 Effects of omeprazole, valinomycin, and ouabain on the exogenous ATP13A2 activity in HEK293 cells. a Effect of ouabain (30 µM) on the TG-sensitive ATPase activity in the ATP13A2-transfected HEK293 cells. (n = 3 independent replicates). NS, $P > 0.05$ vs control (without drug). Statistical significance was determined by two-tailed unpaired Student's t-test. **b** Effect of ouabain (30 µM) on the ATP-dependent $^{86}Rb^{+}$ efflux from lysosomes in the ATP13A2-transfected HEK293 cells. (n = 4-5 independent replicates). Statistical significance was determined by two-tailed unpaired Student's t-test. NS, $P > 0.05$ vs control (without drug). **c** Effects of omeprazole (Ome; 30 µM) and valinomycin (Val; 10 µg/ml) on the TG-sensitive ATPase activity in the ATP13A2-transfected HEK293 cells. In the Ome (HCl), omeprazole (30 µM) was treated with 0.1 N HCl for 20 min. (n = 3-4. independent replicates). Statistical significance was determined by two-tailed unpaired Student's t-test. NS, $P > 0.05$ vs control (without drugs). All data are presented as mean ± SEM. Source data are provided as a source data file.

Supplementary Fig. 4

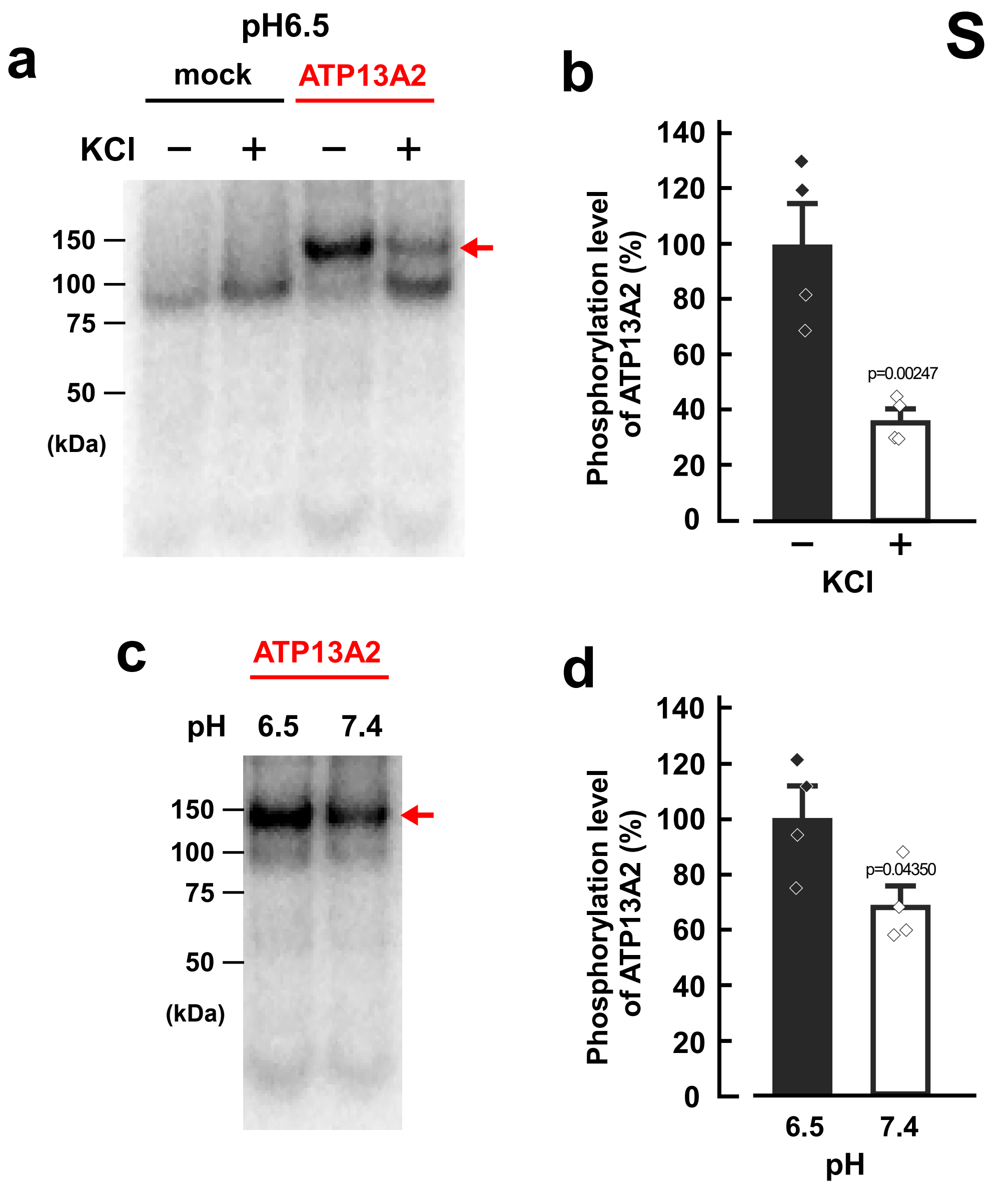

Supplementary Fig. 4 Phosphorylation of ATP13A2 in HEK293 cells. **a** Effects of KCl (20 mM) on the phosphorylation level in the membrane fractions of mock- and ATP13A2-transfected HEK293 cells at pH 6.5. n = 3. The red arrow shows the phosphorylated band (~150 kDa) derived from ATP13A2. **b** The phosphorylated bands of ATP13A2 in **a** were quantified. (n = 4 independent replicates). Statistical significance was determined by two-tailed unpaired Student's t-test. **c** The phosphorylation level at pH 6.5 and 7.4 in the absence of KCl. The membrane fractions of ATP13A2-transfected cells were used. The red arrow shows the phosphorylated band (~150 kDa) derived from ATP13A2. **d** The phosphorylated bands of ATP13A2 in **c** were quantified. (n = 4 independent replicates). Statistical significance was determined by two-tailed unpaired Student's t-test. All data are presented as mean ± SEM. Source data are provided as a source data file.

Supplementary Figure 5

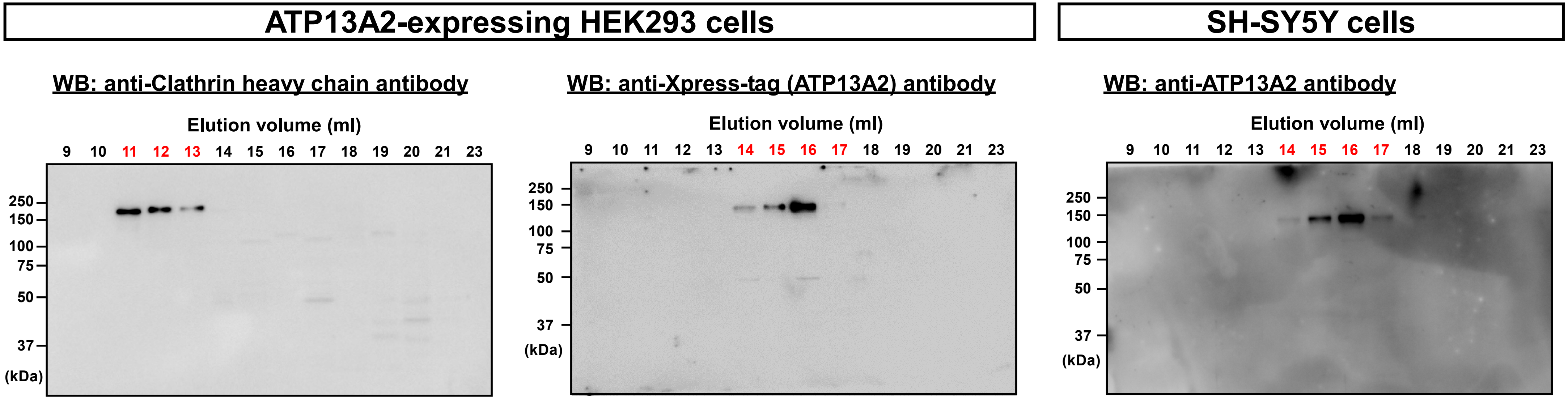

Supplementary Fig. 5 Size-exclusion chromatography profile of exogenous and endogenous ATP13A2. Size-exclusion chromatography was performed with the solubilized membrane fractions of the exogenously ATP13A2-expressing HEK293 cells and the endogenously ATP13A2-expressing SH-SY5Y cells. Typical images of Western blotting using ant-Xpress-tag, anti-ATP13A2, and anti-clathrin heavy chain antibodies were shown. The eluted fractions (elution volumes) of 1 ml were collected. Both exogenous and endogenous ATP13A2 were detected at the same fractions with elution volumes of 14-17 ml, while clathrin heavy chain was detected at fractions with elution volumes of 11-13 ml. It is noted that the molecular size of proteins in the 11-13 ml fractions is higher than in the 14-17 ml fractions. These elusion volumes of ATP13A2 are similar to those of purified monomeric ATP13A2 in the size-exclusion chromatography previously reported in cryo-EM structural studies[16,17].
