# Supplementary material for: Parkinson’s disease-associated ATP13A2/PARK9 functions as a lysosomal H+,K+-ATPase
Source: Nat Commun. 2023 Apr 20;14:2174. doi: 10.1038/s41467-023-37815-z (PMC10119128; doi:10.1038/s41467-023-37815-z)
Supplement: Supplementary file 3 — Reporting Summary [file 41467_2023_37815_MOESM3_ESM.pdf]

nature portfolio

Corresponding author(s): Takuto Fujii, Hideki Sakai

Last updated by author(s): Mar 21, 2023

Reporting Summary

Nature Portfolio wishes to improve the reproducibility of the work that we publish. This form provides structure for consistency and transparency in reporting. For further information on Nature Portfolio policies, see our Editorial Policies and the Editorial Policy Checklist.

Statistics

For all statistical analyses, confirm that the following items are present in the figure legend, table legend, main text, or Methods section.

n/a	Confirmed	
☐	☒	The exact sample size (*n*) for each experimental group/condition, given as a discrete number and unit of measurement
☐	☒	A statement on whether measurements were taken from distinct samples or whether the same sample was measured repeatedly
☐	☒	The statistical test(s) used AND whether they are one- or two-sided<br>*Only common tests should be described solely by name; describe more complex techniques in the Methods section.*
☒	☐	A description of all covariates tested
☐	☒	A description of any assumptions or corrections, such as tests of normality and adjustment for multiple comparisons
☐	☒	A full description of the statistical parameters including central tendency (e.g. means) or other basic estimates (e.g. regression coefficient) AND variation (e.g. standard deviation) or associated estimates of uncertainty (e.g. confidence intervals)
☐	☒	For null hypothesis testing, the test statistic (e.g. *F*, *t*, *r*) with confidence intervals, effect sizes, degrees of freedom and *P* value noted<br>*Give P values as exact values whenever suitable.*
☒	☐	For Bayesian analysis, information on the choice of priors and Markov chain Monte Carlo settings
☒	☐	For hierarchical and complex designs, identification of the appropriate level for tests and full reporting of outcomes
☐	☒	Estimates of effect sizes (e.g. Cohen's *d*, Pearson's *r*), indicating how they were calculated

Our web collection on statistics for biologists contains articles on many of the points above.

Software and code

Policy information about availability of computer code

Data collection	The cDNA sequences were obtained by an ABI PRISM 3500 sequencer (Applied Biosystems).<br>Western blotting was performed with LAS-4000 system (FujiFilm).<br>Immunofluorescence images were visualized by using a Zeiss LSM 780 laser scanning confocal microscope (Zeiss).<br>Size extrusion chromatography was performed with AKTAexplorer 10 XT FPLC system (GE Healthcare).<br>The radioactivity was visualized with Typhoon FLA 9500 (Cytiva).
Data analysis	The bands detected by Western blotting were quantified with ImageJ software e (version: 1.8.0).<br>The intensity of immunofluorescence images was quantified with Zen 3.3 software (Zeiss).

For manuscripts utilizing custom algorithms or software that are central to the research but not yet described in published literature, software must be made available to editors and reviewers. We strongly encourage code deposition in a community repository (e.g. GitHub). See the Nature Portfolio guidelines for submitting code & software for further information.

Data

Policy information about availability of data

All manuscripts must include a data availability statement. This statement should provide the following information, where applicable:

- Accession codes, unique identifiers, or web links for publicly available datasets
- A description of any restrictions on data availability
- For clinical datasets or third party data, please ensure that the statement adheres to our policy

All data are available in the Article, Figures, supplementary materials, and Source data files.

Human research participants

Policy information about studies involving human research participants and Sex and Gender in Research.

Reporting on sex and gender	Female
Population characteristics	Japanese gastric cancer patient (Female, 67 years)
Recruitment	This patient had surgery for treatment of stomach cancer at Toyama University Hospital. Informed consent was obtained from the patient at Toyama University Hospital. There is no self-selection and other biases of this study.
Ethics oversight	The recommendations of the Declaration of Helsinki and the ethics committee approval of the University of Toyama (No. R2017085).

Note that full information on the approval of the study protocol must also be provided in the manuscript.

Field-specific reporting

Please select the one below that is the best fit for your research. If you are not sure, read the appropriate sections before making your selection.

☒ Life sciences ☐ Behavioural & social sciences ☐ Ecological, evolutionary & environmental sciences

For a reference copy of the document with all sections, see nature.com/documents/nr-reporting-summary-flat.pdf

Life sciences study design

All studies must disclose on these points even when the disclosure is negative.

Sample size	No statistical methods were used to predetermine the sample size. All experimental data were obtained from three or more independent experiments to ensure each data point was reproducible.
Data exclusions	No data was excluded in this study
Replication	All experiments were done at least three times. The meaning of "n" in each figure is described in the figure legend in manuscript.<br>All attempts for replication shows similar results.
Randomization	Within each experimental regime, cell populations were assigned randomly to a siRNA, drug or control treatment.
Blinding	The experimentators were blinded towards the code if necessary.

Reporting for specific materials, systems and methods

We require information from authors about some types of materials, experimental systems and methods used in many studies. Here, indicate whether each material, system or method listed is relevant to your study. If you are not sure if a list item applies to your research, read the appropriate section before selecting a response.

Materials & experimental systems

n/a	Involved in the study
	☒ Antibodies
	☒ Eukaryotic cell lines
☒	Palaeontology and archaeology
☒	Animals and other organisms
☒	Clinical data
☒	Dual use research of concern

Methods

n/a	Involved in the study
☒	ChIP-seq
☒	Flow cytometry
☒	MRI-based neuroimaging

Antibodies

Antibodies used

Anti-β-actin antibody (AC-74), A5316, Sigma-Aldrich, Lot 122M4755, 1:5,000 dilution in Western Blotting.
Anti-Xpress-tag antibody, R910-25, Thermo Fisher Scientific, Lot 2478354, 1:5,000 dilution in Western Blotting.
Anti-Na+,K+-ATPase α1-isoform antibody (C464.6), sc-21712, Santa Cruz Technology, Lot G1219, 1:3,000 dilution in Western Blotting.
Anti-phosphorylated α-synuclein antibody (pSyn#64), 015-25191, Fujifilm Wako, Lot SKP4579, 1:100 dilution in immunocytochemistry.
Anti-ATP13A2, # 5879, Cell Signaling Technology, Lot 5879S, 1:1,000 dilution in Western Blotting.
Anti-Lamp2, #49067, Cell Signaling Technology, Lot 40275, 1:100 dilution in immunocytochemistry.
Anti-calnexin, #2679, Cell Signaling Technology, Lot C5C9, 1:100 dilution in immunocytochemistry.
Anti-clathrin heavy chain antibodies, #4796, Cell Signaling Technology, Lot 0003, 1:2,000 dilution in Western Blotting.
Anti-H+,K+-ATPase α-subunit antibody (1H9), D031-3, Medical & Biological Laboratories, Lot 024, 1:5,000 dilution in Western Blotting.
Anti-H+,K+-ATPase β-subunit antibody (2B6), D032-3, Medical & Biological Laboratories, Lot 022, 1:5,000 dilution in Western Blotting.
Anti-α-synuclein antibody (MJFR1), ab138501, Abcam, Lot GR33695272-8, 1:2,000 dilution in Western Blotting.
Alexa Fluor 488-conjugated mouse IgG antibody, ab150109, Abcam, Lot GR3370588-1, 1:100 dilution in immunocytochemistry.
Alexa Fluor 568-conjugated rabbit IgG antibody, ab175692, Abcam, Lot GR3422009-2, 1:100 dilution in immunocytochemistry.
Horse-radish peroxidase-conjugated anti-mouse IgG, AP192, Millipore, Lot 3273149, 1:3,000 dilution in Western Blotting.
Horse-radish peroxidase-conjugated anti-rabbit IgG, AP182, Millipore, Lot 3537617, 1:3,000 dilution in Western Blotting.

Validation

Anti-β-actin antibody (AC-74), Anti-β-actin antibody (AC-74), Anti-Na+,K+-ATPase α1-isoform antibody, Anti-H+,K+-ATPase α-subunit antibody (1H9), Anti-β-subunit antibody (2B6), Anti-clathrin heavy chain antibody are validated in our previous reports; Fujii et al., JBC, 2008, 2009, , FEBS Lett., 2013, BBA-Mol. Basis. Dis., 2018.
Anti-ATP13A2 antibody for human ATP13A2 in Western blotting was validated in this paper using ATP13A2-knockdown SH-SY5Y cells and ATP13A2-knockout HEK293 cells.
Anti-phosphorylated α-synuclein antibody (pSyn#64) is widely used in various reports such as Saito et al., J. Neuropath. Exp. Neurol., 2003, Tsukita et al., Mov. Dis., 2019, Hosokawa et al., Sci. Rep., 2017.
Anti-α-synuclein antibody (MJFR1) is also widely used various reports such as Dohgu et al., Microvascular Res., 2019, Brudek et al., JNC, 2015.

Eukaryotic cell lines

Policy information about cell lines and Sex and Gender in Research

Cell line source(s)

Human embryonic kidney HEK293 cells were kindly provided by Prof. Shinji Asano (Ritsumeikan University).
Human neuroblastoma SH-SY5Y cells (EC94030304-F0) were purchased from DS Pharma Biomedical.

Authentication

None of the cell lines was authenticated by authors.

Mycoplasma contamination

Cell were tested regulatory for mycoplasma contamination and were found to be negative for mycoplasma

Commonly misidentified lines
(See ICLAC register)

No commonly misidentified lines were used in this study.
